# Assessment of Postpartum Depression and Anxiety among Females Attending Primary Health Care Facilities in Qaliubeya Governorate, Egypt

**DOI:** 10.1155/2019/3691752

**Published:** 2019-12-26

**Authors:** Osama M. Wassif, Abdo S. Abdo, Mona A. Elawady, Abeer E. Abd Elmaksoud, Rasha Sh. Eldesouky

**Affiliations:** Community Medicine Department, Faculty of Medicine, Benha University, Banha, Egypt

## Abstract

Postpartum depression (PPD) is a mood disorder that begins after childbirth and usually lasts beyond six weeks; depression is often comorbid with anxiety. The main objectives of this work were to measure the prevalence of postpartum depression and/or anxiety among females in the Qaliubeya governorate to explore the underlying factors of these disorders and find if progesterone level has a role. A crosssectional study was conducted upon 500 postpartum females attending primary health care facilities in the Qaliubeya governorate. Data were collected by an interview questionnaire which included data about sociodemographic, obstetric, and past history and the Arabic version of DASS for assessment of postpartum depression and/or anxiety. The results showed 1.6% of the studied females suffered postpartum depression alone, 10% suffered from anxiety alone, and 21.2% suffered from both. The mean age of female who suffered from comorbid depression and anxiety was significantly (*p*=0.01) higher than the normal group (26.9 and 25.1, respectively), and they had a significantly lower socioeconomic score than the normal ones (31.1 and 34.1, respectively), *p* < 0.05. There was a significant association (*p* < 0.001) between the past history of similar conditions and the current prevalence of postpartum disorders. ROC curve analysis showed that the progesterone level ≤4.6, ≤11.3, and ≤2.8 significantly predict depression alone, anxiety alone, and comorbid diseases, respectively. It was concluded that postpartum depression and/or anxiety affect 32.8% of females in the Qaliubeya governorate. Very low socioeconomic level, lower educational levels, past history of similar conditions, and low progesterone level are the significant predictors.

## 1. Introduction

The major depressive episode (MDE), as defined by the Diagnostic and Statistical Manual (DSM-IV), does not differ in the postpartum period as compared to other times and includes at least 2 weeks of persistent low mood or anhedonia, as well as at least four of the following: increased or decreased appetite, sleep disturbance, psychomotor agitation or retardation, low energy, feeling of worthlessness, low concentration, and suicidal ideation [1].

Depression is often comorbid with anxiety [2]. Postpartum anxiety is a mental health disorder characterized by feeling anxious. Anxiety can occur at any time, but when it occurs in the year after childbirth, it is called postnatal anxiety [3].

Postpartum depression (PPD) is the most common psychological health problem among women, affecting 10%–15% worldwide [4]. Regarding maternal postpartum anxiety, the prevalence rate ranged from 9% to 13% worldwide [5]^.^

The following risk factors are strong predictors of postpartum depression or anxiety: stressful recent life events, poor social support, and a previous history of depression [6]^.^Drop in progesterone contributes to the development of a mood disorder [7]^.^

Postpartum depression and anxiety have many negative consequences on maternal and infant health that are not restricted to infancy, but can also extend into toddlerhood, school age, and even adulthood [8]. Maternal consequences include physical health, psychological health, relationship, and risky behaviors; the infant consequences include anthropometry, physical health, sleep, and motor, cognitive, language, emotional, social, and behavioral development as a conduct disorder in adolescents; and mother-child interactions, include bonding, breastfeeding, and the maternal role [9, 10]. Research supports the idea that a combination of medicine, counseling, support groups, and self-help strategies are the most effective ways to treat depression and anxiety [11].

It has been observed that postpartum depression and anxiety occurrence among women in Egypt have become frequent. However, studies in this area are lacking [12]. So, research on postpartum depression is urgently needed to plan long-term strategies that contribute in avoidance and management of such disorders. So, the aims of this work were to measure the prevalence of postpartum depression and/or anxiety among females in the Qaliubeya governorate to explore the underlying factors of these disorders and find if progesterone level has a role.

## 2. Methodology

### 2.1. Study Design, Population, and Sampling

A comparative crosssectional study was conducted upon 500 postpartum females attending the vaccination clinics at primary health care facilities in the Qaliubeya governorate.

Multistage random sampling technique was used to select the sites, where 3 districts (Kafr Shokr, Khanka, and Shubra Alkhima) were chosen by simple random sampling amongst the 8 districts of the governorate, and then one rural and one urban unit were chosen from each district also by simple random sampling. Females attending the vaccination clinics during their infant vaccination at 2 and 4 months of age were the target population. A minimum sample size of 385 females was calculated using the following equation: *n* = (*Z*^2^ *∗* *P* *∗* *Q*)/(*E*^2^), where *Z* = 1.96, *P* = proportion of postpartum depression according to Vigod et al. [4] = 0.1, *Q*=1 − *p*, and *E* standard error = 0.03. Females fulfilling the inclusion criteria (two or four months postpartum and not suffering any chronic psychiatric diseases) and accepted to participate when invited by the investigators were included. The total number was 500 females.

### 2.2. Data Collection

Data collection was carried out from September 2016 to the end of January 2017 using the following: (A) an Arabic interview questionnaire sheet. It consisted of three parts: part one assessed the sociodemographic characters of the participants including their socioeconomic level (SEL) [13]. Part two included data about their obstetric [14] and past history of similar conditions. Part three included the Arabic version of the Depression Anxiety Stress Scales (DASS-42) according to Moussa et al. [15] who translated the items of English DASS-42 [16, 17] and evaluated the psychometric properties of an Arabic language version to validate it for research use. The Arabic DASS has been proven to discriminate between depression, anxiety, and stress.

DASS-42 is a 42-item questionnaire designed to measure the negative emotional states of depression, anxiety, and stress, with 14 items for each scale, the authors used the questions related to depression (Qs: 3, 5, 10, 13, 16, 17, 21, 24, 26, 31, 34, 37, 38, and 42) and anxiety (Qs: 2, 4, 7, 9, 15, 19, 20, 23, 25, 28, 30, 36, 40, and 41). Respondents were asked to use 4-point severity/frequency scales to rate the extent to which they have experienced each state over the past 2 weeks as follows: 0 = did not apply to me at all, 1 = applied to me some of the time, 2 = applied to me to a good part of time, and 3 = applied to me most of the time. The maximum score is 42 for each disorder. For the depression scale, a score of 0–9 indicates absence of depression, 10–13 mild depression, 14–20 moderate depression, 21–27 severe depression, and a score ≥28 is extremely severe depression. Regarding the anxiety scale, women rated 0–7 were not anxious, 8-9 mild anxiety, 10–14 moderate anxiety, 15–19 severe anxiety, and a score ≥20 indicates extremely severe anxiety [17].

The content and construct validity of the questionnaire were assessed by 3 academic professors (two of public health and a psychiatric one). Face validity was assessed during a pilot study carried upon 30 postpartum females attending the MCH centre in Benha City, required modification in the sheet was done. The results of the pilot study were not included in this work.

Serum progesterone level was measured for all studied females. The venous blood samples were withdrawn by a qualified nurse at the primary health care facilities according to the standards described by the Public Health Ontario [18] and put, stored, and examined by using ELISA Kit (catalog number KA0235,96 assays and version: 02). Tietz [19] performed laboratory investigations at the Department of Clinical Pathology, Benha University Hospital. The filled sheets and blood samples were collected by the researchers at the same time from each participant.

### 2.3. Ethical Consideration

This study was approved by the Research Ethics Committee of the Benha Faculty of Medicine. An administrative permission was taken to interview the target females. Finally, an informed written consent was obtained from all participants. It included data about the title, objectives, methods, benefits, and expected risks and confidentiality of data.

### 2.4. Statistical Analysis

Data were analyzed using STATA/SE version 11.2 for Windows. They were summarized in terms of mean ± standard deviation (SD), median, interquartile range (IQR), and range for quantitative data as appropriate and frequency and percentage for qualitative data. Comparisons between the proportions were carried out by using the chi-square test (*χ*^2^) and Fisher's exact test (FET). Quantitative data were tested for normality using the Kolmogorov–Smirnov test, assuming normality at *p* > 0.05. Student's *t*-test and the one way analysis of variance (ANOVA) were used to compare differences between two and more than two groups, respectively, regarding normally distributed data. However, Mann–Whitney and Kruskal–Wallis tests were used to compare nonparametric data. The Bonferroni method was used to detect differences in pairs. Nonparametric correlations were assessed by Spearman's correlation coefficient (rho). ROC curves were constructed to detect cutoff values of progesterone with optimum sensitivity and specificity in prediction of PP depression and/or anxiety. Stepwise binary logistic regression analysis was run to detect the significant predictors of these disorders. *p* value ≤0.05 was considered significant.

## 3. Results

### 3.1. Basic Characteristics of the Studied Postpartum Females

This study included 500 postpartum females, and their age ranged between 18 and 40 years with a mean value 25.6 years ± 5.07 and 59.6% were from urban areas and 49.2% achieved high education. The duration after birth ranged between 2 and 6 months with an average of 2.9 ± 1.02, the number of family member ≥5 was 67.4%, 89.4% belonged to lower socioeconomic classes while 10.6% were of moderate one, and the score of SEL ranged between 15 and 49 with an average of 33.3 ± 7.06. The gestational age (weeks) of the born infants ranged between 34 and 38 weeks with an average of 34.7 ± 2.29, in which 60.8% were delivered by caesarean section; about half of them were females (50.2%). The order of the born child ranged between 1 and 6 with median 2. Majority of women (88.5%) took iron during pregnancy as 76.4% of them had anemia during pregnancy. 15.6% of the studied females had a past history of postpartum depression and/or anxiety. Both depression and anxiety scores ranged from 0 to 37 with mean values of 5.35 ± 28.6 and 5.95 ± 26.45, respectively, and the median and interquartile range (IQR) was 4 (1–9) and 4 (1–8), respectively. Progesterone level ranged 0.02–369. The average was 49.2 ± 63.7, and the median and IQR was 25.0 (3.02–68.5) (Table 1).

### 3.2. Prevalence and Severity of Postpartum Depression and Anxiety

**Figure 1:**
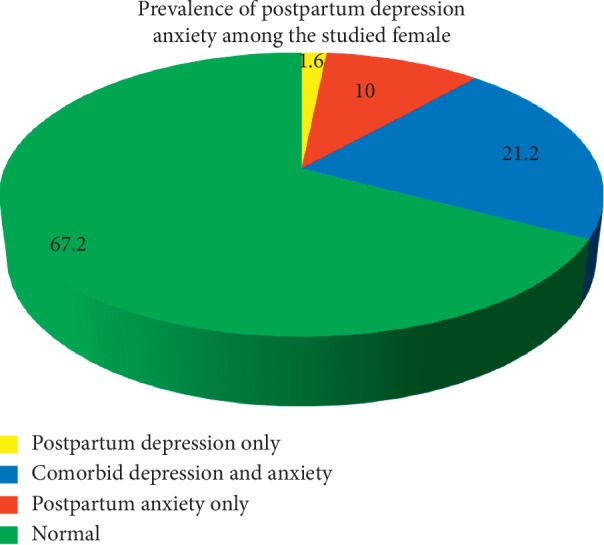
Pie chart showing the prevalence of postpartum depression and anxiety among the studied postpartum females. For the depression scale, a score of 0–9 indicates absence of depression and ≥10 indicates depression regardless the degree. Regarding the anxiety scale, women rated 0–7 were not anxious and ≥8 anxiety regardless the degree [17]. Accordingly, women were classified either depressed only, anxious only, or both.

**Figure 2:**
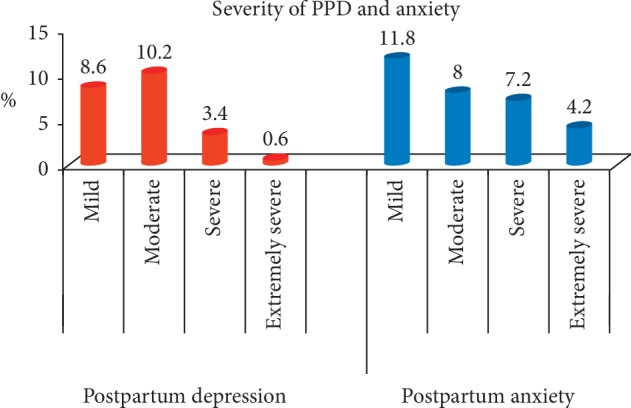
Bar chart showing the severity of postpartum depression and anxiety among the studied postpartum females.

The results showed that 1.6% of the studied females suffered postpartum depression alone, 10% suffered from anxiety alone, and 21.2% suffered from both (Figure 1). Considering severity, 8.6%, 10.2%, 3.4%, and 0.6% suffered from mild, moderate, severe, and extremely severe postpartum depression, respectively. 11.8%, 8.0%, 7.2%, and 4.2% suffered mild, moderate, severe, and extremely severe postpartum anxiety, respectively (Figure 2).

### 3.3. Association between Postpartum Depression and Anxiety and the Studied Variables

The mean age of female who suffered from comorbid depression and anxiety was significantly (*p*=0.01) higher than the normal group (26.9 and 25.1, respectively). Also, they had a significantly lower socioeconomic score than the normal ones (31.1 and 34.1, respectively), *p* < 0.05. Twenty-two percent and 32.1% of females with anxiety only and comorbid disorders had a past history of similar conditions compared to 9.8% of the normal group and 0% of the depression only group. This difference was statistically significant (*p* < 0.001) (Table 2).

### 3.4. Progesterone Level among the Studied Females

**Figure 3:**
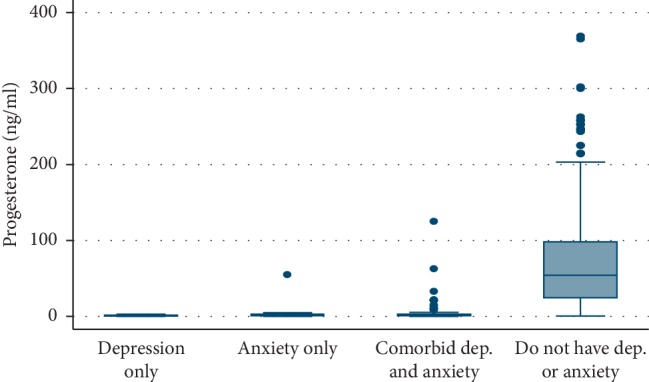
Box plot showing median and IQR of progesterone level according to the prevalence of PPD and anxiety among the studied females, *p* < 0.001 (HS). Median (IQR): 1.23 (0.63–1.83), 2.05 (1.02–3.06), 2.02 (1.02–3.02), and ^cba^ 54.6 (24.15–98.3). a: significant difference compared to comorbid group; b: significant difference compared to anxiety only group; c: significant difference compared to depression only group.

Figure 3 shows that the median values of progesterone level were significantly (*p* < 0.001) lower among females with depression alone, anxiety alone, and comorbid diseases than the normal group (1.23, 2.05, 2.02, and 54.6, respectively).

**Figure 4:**
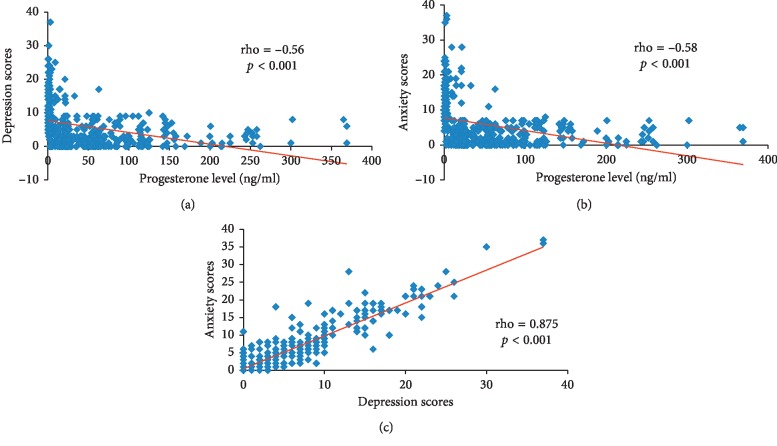
Scatter graphs (a) and (b) show significant negative correlations between progesterone level and depression scores and anxiety scores. Graph (c) shows a significant positive correlation between depression and anxiety scores.

Scatter graphs (Figures 4(a) and 4(b)) show significant negative correlations between progesterone level and depression scores (rho = −0.56, *p* < 0.001) and anxiety scores (rho = −0.58, *p* < 0.001).  Graph 4(c) shows a significant positive correlation between depression and anxiety scores (rho = 0.875, *p* < 0.001).

The results also revealed a positive significant correlation between age and anxiety scores (rho = 011, *p*=0.01) and negative correlation between SE score and both depression scores (rho = −0.25, *p* < 0.001) and anxiety scores (rho = −0.20, *p* < 0.001).

**Figure 5:**
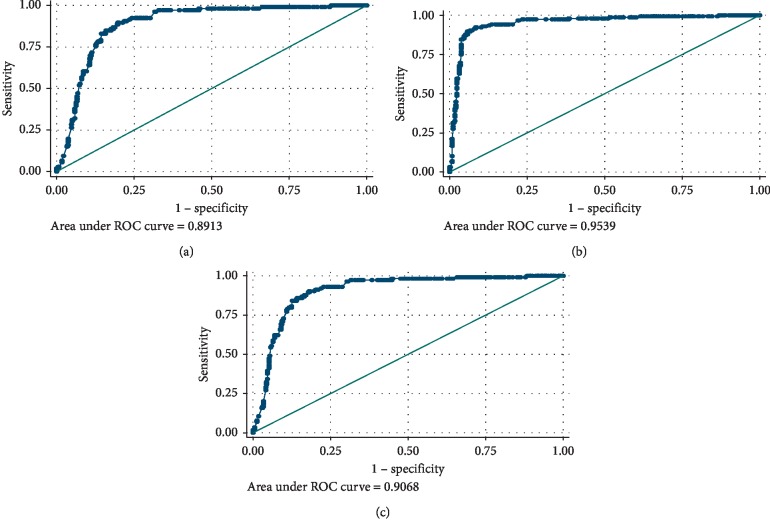
ROC curves showing AUC of progesterone level in prediction of depression and/or anxiety: (a) depression, (b) anxiety, and (c) comorbid group.

ROC curve analysis showed that progesterone cutoff values of ≤4.6, ≤11.3, and ≤2.8 significantly predict depression alone, anxiety alone, and comorbid diseases, respectively. The areas under the curve (AUCs) were 0.9, 0.95, and 0.9, respectively (Figure 5) (Table 3).

### 3.5. Predictors of PPD and/or Anxiety

Binary logistic regression analysis showed that very low SEL, past history of similar conditions, and progesterone level ≤4.6 are the significant predictors of PPD alone (ORs = 4.7, 2.56, and 1.11, respectively), *p* < 0.05 for all. While lower educational levels, past history of similar conditions, and low progesterone level are significant (*p* < 0.05) predictors for both anxiety alone and comorbid disorders (Table 4).

## 4. Discussion

This study has been conducted upon postpartum females aged 18–40 years, with the benefits of investigating this vulnerable age group using a standardized questionnaire. Moreover, literature on PPD and anxiety in Egypt has revealed lack of the impact of progesterone levels on these disorders.

The current results showed that 1.6% and 10% of the studied females suffered from isolated postpartum depression (PPD) and anxiety, respectively, and these figures are less than those reported by Miller et al. [20] who conducted a study on 325 mothers in Melbourne, Australia, using DASS-21. They found that 19% and 13% of females were depressed and anxious, respectively. Differences might occur because most of the females did not have only depression or anxiety, but in most of the times combined with other mental disorders or stress.

Other studies on PPD and anxiety prevalence in Egypt showed higher figures than the current work. Salem et al. [12] in their crosssectional study at Sohag University Hospital reported that the prevalence of PPD was 7% which reflected the highest prevalence among the regions in Egypt. This could be due to other cofactors such as regional culture, attitudes towards females, or the tool they used (Edinburgh Postnatal Depression Scale, EPDS).

In Canada, Fairbrother et al. [21] in their work upon 115 women stated that the prevalence of anxiety in the early postpartum period was17%, while the percentage of depression was 4.8%.

Peñacoba-Puente et al. [22], in their study revealed that postpartum symptoms of depression and anxiety were all significantly correlated with each other. This agrees with our finding that 21.2%, about two thirds of the suffering females, have comorbid depression and anxiety. These results are higher than those noted by Falah-Hassani et al. [23] in their study in British Columbia which included 522 women using EPDS. They found that comorbid depression and anxiety was 13%.

The current work showed the percentages of the severity of PPD (8.6% mild, 10.2% moderate, 3.4% severe, and 0.6% extremely severe) and anxiety (11.8% mild, 8.0% moderate, 7.2% severe, and 4.2% extremely severe). These figures are less than those stated by Deltsidou et al. [24] who investigated 480 postpartum women in Athens except for mild anxiety. They concluded that depression levels in their sample were 13.1% mild, 19.3% moderate, 10% severe, and 21.3% were extremely severe, while anxiety grades were 2.5% mild, 21.9% moderate, 19.4% severe, and 31.9% extremely severe. The difference between both results may be due to different socioeconomic characters of the target populations. Moreover, Deltsidou et al. used the DASS-21 scale.

Based on the present results, the mean age of females who suffered from comorbid depression and anxiety was significantly (*p*=0.01) higher than the normal group. These findings disagree with the results obtained by Yelland et al. [25] who studied 8597 postpartum women in Victoria and South Australia. They explained their results that older mothers acquire high levels of maturity and life experience that enable them to cope with the emotions associated with motherhood more than younger ones. Diversity of the current study may be due to accumulation of stressors originating from different environmental conditions, lower socioeconomic level, and burden of responsibilities of many children for older mothers.

The investigators have found that females who had a positive past history of similar conditions were more prone to these problems. These results are in agreement with those of Biaggi et al. [26] in their systematic review, where a meta-analyses of 97 studies were carried out. They reported that the recurrence or persistence of depression or anxiety from pregnancy to the postpartum period is noted in most of the females who had a previous history.

One of this work's objectives was to investigate the role of progesterone level in the occurrence of PPD and/or anxiety. It was revealed that lower progesterone levels are significant predictors of these disorders. This agrees with previous findings by Yim et al. [27] who concluded that women with higher levels of postpartum progesterone may experience lower rates of PPD symptoms. Their conclusion was based on the systematic literature search conducted in PubMed and PsycINFO using 3,597 records.

These findings are also supported by those results of a crosssectional study carried out by Peñacoba-Puente et al. [22] up on 209 women in Madrid, Spain, by using the Beck Depression Inventory II. They said that there was a significant negative correlation between progesterone level and depression scores. Moreover, Skalkidou et al. [28] reported that the peak in postpartum depressive symptoms coincides with the most rapid decline in progesterone level and its metabolite allopregnanolone.

Regarding the role of synthetic progesterone, Fitelson et al. [29] stated that synthetic progesterone not only has no role in preventing the development of postpartum depression but also has a negative effect on maternal mood when used as a contraception.

The present study has investigated the predictors of PPD and/or anxiety. It was found that low SEL is a significant predictor of isolated postpartum depression. This is consistent with Dolbier et al. [30] who enrolled 433 White and African American women in his study. Finally, Stewart et al. [31] who investigated 583 women in Malawi found that women with more years of schooling are more likely to experience anxiety symptoms. This is in disagreement with the current study findings where higher levels of education were found to be protective from postpartum anxiety and comorbid disorders. This different conclusion by Stewart et al. may be due to other cofactors of anxiety among highly educated mothers such as job conditions.

The main limitation of this work is as all crosssection studies, it cannot state causation but only association.

## 5. Conclusion

Postpartum depression and/or anxiety affect about one third (32.8%) of females in the Qaliubeya governorate. Very low SEL, lower educational levels, past history of similar conditions, and low progesterone level are the significant predictors.

## 6. Recommendations

Longitudinal studies are recommended to track different variables prepartum, during pregnancy, and postpartum and to assess stressful life events not studied in this work as domestic violence. Moreover, incidence could be calculated.

## Figures and Tables

**Table 1:** Basic characteristics of the studied postpartum females.

Variable	No. (*n* = 500)	%
Age (yrs)		
Mean ± SD (range)	25.6 ± 5.07 (18–40)	

Residence		
Rural	97	19.4
Urban	298	59.6
Semiurban	105	21.0

Educational level		
Read and write, primary	104	20.8
Preparatory and secondary	150	30
High^*∗*^	246	49.2

Number of months after the baby's birth		
Mean ± SD (range)	2.9 ± 1.02 (2–6)	

Number of family member		
<5	163	32.6
≥5	337	67.4

Socioeconomic status		
Very low	27	5.4
Low	420	84.0
Moderate	53	10.6

SEL score†		
Mean ± SD (range)	33.3 ± 7.06 (15–49)	
Gestational age (w)		
Mean ± SD (range)	34.7 ± 2.29 (4–38)	

Mode of delivery		
NVD	196	39.2
CS	304	60.8

Sex of the born child		
Female	251	50.2
Male	249	49.8

Order of the born child		
Median (range)	2 (1–6)	

Iron intake during pregnancy		
No	58	11.7
Yes	442	88.5

Anemia during pregnancy		
No	118	23.6
Yes	382	76.4

Past history of similar conditions		
No	421	84.4
Yes	78	15.6

Depression score		
Mean ± SD (range)	5.9 ± 6.3 (0–37)	
Median (IQR)	4 (1–9)	

Anxiety score		
Mean ± SD (range)	5.95 ± 6.45 (0–37)	
Median (IQR)	4 (1–8)	

Progesterone levels		
Mean ± SD (range)	49.2 ± 63.7 (0.02–369)	
Median (IQR)	25.0 (3.02–68.5)	

^*∗*^High: institute, college, or postgraduate; † according to (El Gilany et al., 2012) [13].

**Table 2:** Association between postpartum depression and anxiety and the studied variables.

Variable	Depression only (no. = 8)		Anxiety only (no. = 50)		Comorbid depression and anxiety (no. = 106)		Do not have depression or anxiety (no. = 336)		Fisher's exact test
	No.	%	No.	%	No.	%	No.	%	
Age (mean ± SD)	24.8 ± 5.72		26.3 ± 5.13		26.9 ± 5.35		^a^25.1 ± 4.89		*F* = 3.57 *p*=0.01(S)

Residence									
Rural	1	12.5	9	18	22	20.8	65	19.4	0.2
Urban	6	75	27	54	54	50.9	211	62.8	
Semiurban	1	12.5	14	28	30	283	60	17.9	

Educational level									
Read and write, primary	0	0	9	20	26	24.5	68	20.2	0.57
Preparatory and secondary	4	50	27	34	33	31.1	96	28.6	
High	4	50	14	46	47	44.3	172	51.2	

Number of months after the baby's birth (mean ± SD)	3.25 ± 1.03		3.04 ± 1.01		2.9 ± 1		2.85 ± 1.03		*F* = 0.88 *p*=0.45

Number of family member									
<5	3	37.5	19	38	39	36.8	102	30.4	0.46
≥5	5	62.5	31	62	67	63.2	234	69.6	

Socioeconomic status									
Very low	0	0	2	4	13	12.3	12	3.6	0.07
Low	8	100	42	84	84	79.3	286	85.1	
Moderate	0	0	6	12	9	8.5	38	11.3	

SE score (mean ± SD)	32.8 ± 5.84		32.9 ± 7.05		31.1 ± 7.84		^a^34.1 ± 6.7		*F* = 4.92 *p*=0.002(S)

Gestational age (w) mean ± SD	34.25 ± 1.98		34.8 ± 1.76		34.7 ± 1.72		34.65 ± 2.52		*F* = 0.15 *p*=0.93

Mode of delivery									
NVD	1	12.5	20	40	45	42.5	130	38.7	0.43
CS	7	87.5	30	60	61	57.6	206	61.3	

Sex of the born child									
Female	3	37.5	22	44	49	46.2	177	52.7	0.42
Male	5	62.5	28	56	57	53.8	159	47.3	

Order of the born child									
Median (IQR)	2 (1–3)		2 (1–3)		2 (1–3)		2 (1–3)		kw = 1.45 *p*=0.69

Iron pills given during pregnancy									
No	0	0.0	7	14.0	12	11.3	39	11.7	0.85
Yes	8	100.0	43	86	94	88.7	295	88.3	

Anemia during pregnancy									
No	2	25.0	11	22	20	18.9	85	25.3	0.58
Yes	6	75.0	39	78	86	81.1	251	74.7	

Past history of similar conditions									
No	8	100.0	39	78.0	72	67.9	303	90.2	<0.001 (HS)
Yes	0	0.0	11	22.0	34	32.1	33	9.8	

*F*: one way analysis of variance (ANOVA); kw: Kruskal–Wallis test; HS: highly significant; S: significant; a: significant difference compared to comorbid depression and anxiety.

**Table 3:** ROC curve for the performance of progesterone level in prediction of depression, anxiety, and comorbid depression and anxiety.

Diagnosis	Cutoff	Sensitivity (%)	Specificity (%)	PPV (%)	NPV (%)	Accuracy (%)	AUC (95%CI)	*p* value
Depression	≤4.6	85.9	85.2	63.2	95.4	85.4	0.891 (0.877 to 0.935)	**<0.001 (HS)**

Anxiety	≤11.3	92.3	91.6	83.2	96.3	91.8	0.954 (0.877 to 0.935)	**<0.001 (HS)**

Comorbid depression and anxiety	≤2.8	84.9	89.1	67.7	95.6	88.2	0.906 (0.859 to 0.922)	**<0.001 (HS)**

**Table 4:** Binary logistic regression analysis for the predictor of postpartum depression, anxiety, and comorbidity.

Variable	Postpartum depression		Postpartum anxiety		Postpartum comorbidity	
	OR (95% CI)	*p* value	OR (95% CI)	*p* value	OR (95% CI)	*p* value
Educational level: Read and write/primary Preparatory and secondary High			4.5 (1.75–11.1) 3.1 (1.16–8.3) Reference	**0.002 (S)** **0.024 (S)**	2.5 (1.2–5.3) 2.1 (0.97–4.55) Reference	**0.012 (S)** **0.059**

Socioeconomic status (SES) Very low Low Moderate	4.7 (1.2–20) 2.63 (0.88–3.03) Reference	**0.025 (S)** 0.086				

Past history of similar conditions (yes vs. no)	2.56 (1.32 to 4.94)	**0.005 (S)**	5.35 (2.25 to 12.73)	**<0.001 (HS)**	3.26 (1.7 to 6.25)	**<0.001 (HS)**

Progesterone levels ≤4.6	1.11 (1.07 to 1.15)	**<0.001 (HS)**	1.18 (1.14–1.22)	**<0.001 (HS)**	0.91 (0.88 to 0.93)	**<0.001 (HS)**

OR: odds ratio; CI: confidence interval.

## Data Availability

The data used to support the findings of this study are available from the corresponding author upon request.
